# Mutual Effect Modification between Insulin Resistance and Endothelial Dysfunction in Predicting Incident Heart Failure in Hypertensives

**DOI:** 10.3390/biomedicines11082188

**Published:** 2023-08-03

**Authors:** Maria Perticone, Raffaele Maio, Simona Gigliotti, Ermal Shehaj, Alfredo Francesco Toscani, Antonella Capomolla, Ginevra Fabiani, Angela Sciacqua, Francesco Perticone

**Affiliations:** 1 Department of Medical and Surgical Sciences, Magna Graecia University of Catanzaro, 88100 Catanzaro, Italy; a.toscani91@gmail.com (A.F.T.); sciacqua@unicz.it (A.S.); perticone@unicz.it (F.P.); 2 Geriatrics Unit, Azienda Ospedaliero-Universitaria Renato Dulbecco, 88100 Catanzaro, Italy; raf_maio@yahoo.it; 3 Department of Health Sciences, Magna Graecia University of Catanzaro, 88100 Catanzaro, Italy; simona_gigliotti@yahoo.it; 4 Cardiology and CICU Unit, Giovanni Paolo II Hospital, 88046 Lamezia Terme, Italy; shehajermal@gmail.com; 5 Don Mottola Medical Center, 89862 Drapia, Italy; antok96@gmail.com; 6 Department of Experimental and Clinical Medicine, University of Florence, 50121 Florence, Italy; g.fabiani@unifi.it

**Keywords:** heart failure, insulin resistance, endothelial dysfunction, hypertension, cardiovascular risk factors

## Abstract

Insulin resistance and endothelial dysfunction are associated with heart failure (HF). Our objective was to investigate whether endothelial dysfunction and insulin resistance are independent predictors of incident HF and if a possible interaction exists between them. We enrolled 705 white never-treated hypertensives. Endothelium-dependent vasodilation was investigated by intra-arterial infusion of acetylcholine. During the follow-up [median: 117 months (range: 31–211)], we documented 223 new cases of HF (3.3 events/100 patient-years). We stratified the study population into progressors and non-progressors; progressors showed an older age and a higher prevalence of females, as well as higher mean values of baseline glucose, insulin, homeostasis model assessment (HOMA), creatinine, and high-sensitivity C-reactive protein (hs-CRP), whereas the estimated glomerular filtration rate (e-GFR) and endothelium-dependent vasodilation were lower. In the multiple Cox regression analysis, serum hs-CRP (HR = 1.362, (95% CI = 1.208–1.536), HOMA (HR = 1.293, 95% CI = 1.142–1.465), maximal acetylcholine (Ach)-stimulated forearm blood flow (FBF) (100% increment, HR = 0.807, 95% CI = 0.697–0.934), and e-GFR (10 mL/min/1.73 m^2^ increment, HR = 0.552, 95% CI = 0.483–0.603) maintained an independent association with incident HF. HOMA and endothelial dysfunction interact between them in a competitive manner (HR = 6.548, 95% CI = 4.034–10.629), also showing a mutual effect modification. Our findings demonstrate that both endothelial dysfunction and HOMA are independent and strong predictors of incident HF in hypertensives, these two risk factors interact between them with a competitive mechanism.

## 1. Introduction

Heart failure (HF), due to both structural and functional cardiac alterations with associated neurohormonal activation, is a complex clinical syndrome characterized by diminished quality of life, high mortality, and recurrent and expensive hospital admission [1]. For these reasons, in addition to population aging and HF-associated comorbidities, HF represents a major public health concern [2,3]. Thus, in this context, it is possible to affirm that both ischemic heart disease [4] and type-2 diabetes mellitus [5] represent major clinical conditions involved in the appearance and progression of HF. Thus, the early identification of possible predictors of incident HF may be considered an interesting and important strategy to reduce HF-related morbidity and mortality as well as healthcare costs.

Vascular endothelium is essential for the regulation of vascular tone and the maintenance of vascular homoeostasis, and is an active autocrine, paracrine, and endocrine organ [6]. Dysfunctional endothelium is one of the principal determinants of microvascular alterations by shifting the physiological vascular equilibrium towards vasoconstriction with subsequent organ ischemia, inflammatory and procoagulant state, and proliferation of smooth muscle cells [7]. Endothelial dysfunction is considered the earliest pathophysiologic step in the atherosclerotic process [7,8], and it is an established independent and powerful prognostic factor for adverse cardiovascular outcomes in the different settings of patients [9,10], appearance of new diabetes [11], and incident HF [12]. It has been demonstrated that abnormal endothelium may contribute to HF appearance with different pathogenetic mechanisms (i.e., the reduction of epicardial and small coronary vessels vasoreactivity, the increase of cardiac post-load, myocardial oxidative stress, and fibrotic process [13,14,15]). Furthermore, additional evidence demonstrated that patients with chronic HF, either with reduced or preserved ejection fraction [14,15], have endothelial dysfunction. Thus, HF-related endothelial dysfunction may be considered, at the same time, a marker of heart decompensation.

Thus, the aim of this study was to investigate the possible interaction between endothelium-dependent vasodilation (tested by pharmacologic stimulation of muscarinic receptor) and insulin-resistance state (evaluated by the HOMA-index) in predicting incident HF in a very large and well-characterized group of hypertensive patients. In this paper, contrary to what is already known, we have chosen insulin resistance instead of overt type-2 diabetes mellitus because this condition occurs many years earlier.

## 2. Materials and Methods

From a large cohort of hypertensive patients participating in the CATAnzaro MEtabolic RIsk factors (CATAMERI) study, we recruited a total of 705 Caucasian patients [358 men and 347 women aged 22–73 years (mean age 48.4 ± 10.5 years)], with systolic blood pressure (SBP) ≥ 140 mmHg and/or diastolic blood pressure (DBP) ≥ 90 mmHg. Individuals came to our tertiary University Center directly or were referred by general practitioners for detection or investigation of cardiovascular risk factors. All patients underwent physical examination, review of their medical history, and anthropometrical evaluation: weight, height, and body mass index (BMI). We excluded patients with secondary forms of hypertension, previous cardiovascular events, rheumatic and non-rheumatic valvular heart disease, prosthetic valves, cardiomyopathies, type-2 diabetes mellitus defined as HbA1c ≥ 6.5% or fasting plasma glucose ≥ 126 mg/dL, chronic kidney disease defined as an estimated glomerular filtration rate (e-GFR) < 60 mL/min/1.73 m^2^, malignant diseases, liver and peripheral vascular disease, thyroid disorders or those taking drugs affecting thyroid function, and HF defined according to both clinical and echocardiographic findings. 

The CATAMERI study was submitted and approved on 17 October 2012 (approval number: 2012.63) by the Ethics Committee of the Azienda Ospedaliero-Universitaria Mater Domini of Catanzaro. The investigation conforms with the principles outlined in the Declaration of Helsinki. All the participants gave their informed written consent to study participation.

### 2.1. Blood Pressure Measurements 

After a preliminary blood pressure (BP) measurement in both arms to exclude a possible difference between them, the readings of clinic BP were obtained (according to the current guidelines at the time of the evaluation) after 5 min of quiet rest. A minimum of three BP readings were taken on three separate occasions at least two weeks apart. Systolic and diastolic BP were measured by a standard sphygmomanometer at the first appearance (Phase I) and the disappearance (Phase V) of Korotkoff sounds. Baseline BP values represent the average of the last two of the three consecutive measurements obtained at an interval of 3 min. The diagnosis of hypertension was obtained based on the values of clinic systolic BP ≥ 140 and/or diastolic ≥ 90 mm Hg, respectively. Secondary forms of hypertension were excluded by systematic testing according to a standard clinical protocol, which included laboratory measurements of aldosterone, plasma renin activity, Doppler studies of the renal arteries, and/or renal scintigraphy or renal angiography.

### 2.2. Laboratory Evaluations

At the first eligibility visit, all laboratory measurements were performed after a fasting period of at least 12 h. Total cholesterol, low-density lipoprotein (LDL) cholesterol, high-density lipoprotein (HDL) cholesterol, triglyceride, and fasting glucose were measured using the standard methods (Roche Diagnostics GmbH, Mannheim, Germany, and Glucose Analyzer, Beckman Coulter SpA, Milan, Italy). Serum creatinine was measured by an automated technique based on the measurement of Jaffe chromogen and the URICASE/POD method (Boehringer Mannheim, Mannheim, Germany) implemented in an auto-analyzer. Values of e-GFR were calculated using the equation proposed by investigators in Chronic Kidney Disease Epidemiology (CKD-EPI). High-sensitivity C-reactive protein (hs-CRP) was measured by a turbidimetric immunoassay (Behring, Montgomery, PA, USA).

### 2.3. Insulin-Resistance

Plasma insulin was determined in duplicate by a highly specific radioimmunoassay. *Insulin resistance* was estimated by the homeostasis model assessment (HOMA) from the fasting glucose and insulin concentrations according to the equation: HOMA = [insulin (μU/mL × glucose (mmol/L)]/22.5 [16].

### 2.4. Vascular Function Evaluation

Vascular function assessments were obtained at the time of the first observation by the examiners that were unaware of the patient’s clinical and laboratory parameters. After overnight fasting, all studies started at 09:00 A.M. in a quiet and comfortable air-conditioned room, with the patients in the supine position. The patients were invited to continue their regular diet, whereas caffeine and alcohol were stopped 24 h before the study. Vascular wall reactivity was evaluated using a plethysmography method according to the protocol initially described by Panza [17] and subsequently employed by us [9,11,12,18]. All patients underwent the measurement of forearm blood flow (FBF) and BP during intra-arterial infusion of saline, sodium nitroprusside (SNP), and acetylcholine (ACh) at increasing doses. To reach a stable baseline before data collection, all subjects rested 30 min after artery cannulation; the measurements of FBF and vascular resistance (VR) were repeated every 5 min until stable. A dose-response curve to intraarterial ACh infusions (7.5, 15, and 30 μg/min, each for 5 min) and SNP infusions (0.8, 1.6, and 3.2 μg/min, each for 5 min) was used to assess endothelium-dependent and endothelium-independent vasodilations, respectively. To avoid forearm volume modification, we maintained an infusion rate of 1 mL/min. The sequence of administration of ACh and SNP was randomized to avoid any bias related to the order of drug infusion. Forearm VR, expressed in arbitrary U, was calculated by dividing the mean BP by FBF. For the present study, the maximal response to ACh was considered for statistical analysis.

### 2.5. Follow-Up and Incident Heart Failure

According to current guidelines, all patients were treated to reduce clinic BP < 140/90 mmHg using the standard lifestyle and pharmacological treatment. Angiotensin-converting enzyme (ACE)-inhibitors, angiotensin II receptor antagonists, calcium channel blockers, diuretics, β-blockers, and α-blockers were used alone or in various combinations. 

To improve the long-term follow-up, we have planned periodic control visits in the outpatient clinic at least every six months. In addition, a questionnaire was also mailed to family physicians, and the patients who had missed the clinical evaluation were contacted periodically by phone. 

Incident HF cases were confirmed according to the following criteria: signs and symptoms of dyspnea on exertion, difficulty exercising, and edema; echocardiographic or other imaging tests to evaluate cardiac structure and function, particularly the ejection fraction to define HF with reduced (HF-rEF) or preserved (HF-pEF) left ventricular function; and natriuretic peptides. 

We stratified the study population into two groups (progressors and non-progressors) according to the development of HF or not, respectively.

### 2.6. Statistical Analysis 

The differences between clinical and biological data were tested by one-way ANOVA and unpaired Student’s *t* test for continuous variables, and the X^2^ test for categorical variables. Dose responses to ACh and SNP infusion were compared by ANOVA for repeated measurements and, when the analysis was significant, Tukey’s test was applied.

We reported the event rate as the number of events per 100 patient-years based on the ratio of the number of events observed to the total number of patient-years of exposure up to the terminating event or censor. For non-progressor patients, the date of censor was that of the last contact. The Kaplan-Meier product-limit method was used to estimate survival curves, which were then compared using the Mantel (logistic-rank) test. 

The prognostic effect on the incident HF of endothelial function (expressed as the maximal ACh-stimulated FBF) and other risk factors was analyzed by univariate and multiple Cox regression analyses. The tested covariates included age, gender, smoking, serum cholesterol, SBP, BMI, HOMA, hs-CRP, and e-GFR. In this analysis, we included only the HOMA index to avoid a possible colinearity with both fasting glucose and insulin. Proportional hazards were assessed both by visual inspection and by the log-log method for categorical variables, whereas for continuous variables, the proportional risk assumption was tested by relating the Schoenfeld residuals of the Cox analysis with the survival time. The multiple Cox regression model was constructed by including all variables reaching the statistical significance at univariate Cox regression analysis, allowing us to construct a Cox model of adequate statistical power (at least 10 events for each variable in the final model). Data were expressed as hazard ratio (HR), 95% confidence interval (CI), and *p* value. The maximal ACh-stimulated FBF and HOMA were tested either as continuous variables or by tertiles. 

The mutual effect modifications by altered maximal ACh-stimulated FBF [under or above the median (252%)] and HOMA [above or under the median (3.1)] on the HF incidence was investigated by simultaneously including the following variables into the same multiple Cox model (Model 1): FBF (1 = under the median; 0 = above the median), HOMA (1 = above the median; 0 = under the median), and their interaction term (FBF × HOMA). These mutual effect modifications were also tested in a Cox model by adjusting for age, gender, smoking, SBP, BMI, cholesterol, and antihypertensive drugs (Model 2). The risks for incident HF were calculated by the standard linear combination method expressed as HR (95% CI) with *p* values.

Results are reported as means ± SD or as percentage frequency, and the significance was assumed for *p* value ≤ 0.05. 

## 3. Results

In Table 1, we reported the baseline characteristics of progressors and non-progressors. Progressors were older with a higher prevalence of females, and showed higher mean values of baseline glucose, insulin, HOMA, creatinine, and hs-CRP, whereas e-GFR values were lower. No statistically significant differences were observed between the groups in BMI, smoking habit, SBP and DBP, heart rate, lipid profile, and basal FBF. The highest response ACh-stimulated FBF was significantly lower in progressors than in non-progressors (221 ± 128 vs. 328 ± 189%; *p* < 0.0001), whereas no significant differences were observed in the maximal vasodilation induced by SNP (312 ± 114 vs. 318 ± 111%; *p* = 0.584). At enrollment, none of the patients had been treated with antihypertensive drugs. The baseline BP mean values in the study population were 149.2/91.0 + 16.9/11.6 mmHg, with a little but not significant difference in SBP between the two groups (150.3 + 16.1 vs. 148.7 + 17.3 mmHg). As recommended by the current guidelines, we treated all patients to reduce clinical BP < 140/90 mmHg using both lifestyle modifications and pharmacological treatment. ACE-inhibitors, angiotensin II receptor antagonists, calcium channel blockers, diuretics, β-blockers, and α-blockers were used alone or in different associations without significant differences between the groups (Table 1). The percentage of patients reaching the recommended BP target did not significantly differ between the groups (65.1 vs. 65.9% in the progressors and non-progressors patients, respectively).

### 3.1. Vascular Function

Intra-arterial ACh administration caused a significant dose-dependent increase in FBF and a decrease in VR in the whole study population. The FBF increments from basal (3.36 ± 0.66 mL × 100 mL tissue^−1^ × min^−1^) at the three incremental doses were 1.94 ± 1.22 (+57.7%), 5.46 ± 3.56 (+162.5%), and 10.43 ± 6.31 mL × 100 mL tissue^−1^ × min^−1^ (+310.4%), respectively. At the highest dose of ACh (30 μg/min), FBF increased to 13.79 ± 3.97 mL × 100 mL tissue^−1^ × min^−1^ and VR decreased to 10.16 ± 4.94 U. Remarkably, a significant difference in the maximal ACh-stimulated FBF (221 ± 128 vs. 328 ± 189 mL × 100 mL tissue^−1^ × min^−1^) was observed by stratifying the study population into progressors and non-progressors (Table 1). 

Similarly, we observed a significant increase in FBF (the maximal increment from the basal, +316%) and a decrease in VR (−71%) after SNP infusion, without significant differences between the groups (Table 1). Given the very low doses of vasoactive substances, their intra-arterial infusion caused no changes in both BP and heart rate; in fact, vasoactive substances were infused at very low doses without systemic hemodynamic effects.

### 3.2. Follow-Up and Incident Heart Failure

**Figure 1:**
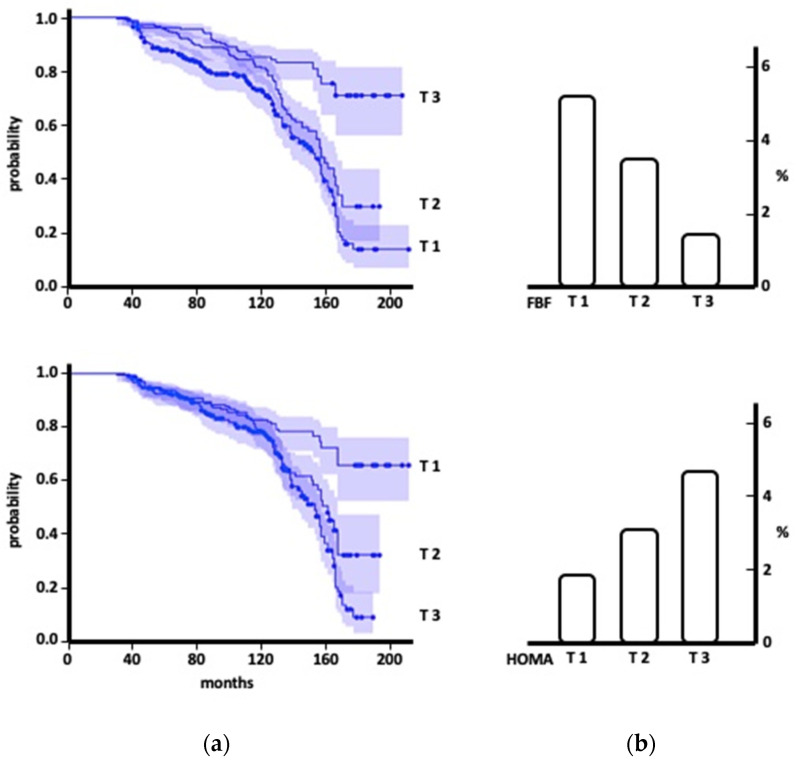
Survival curves for incident heart failure in hypertensive patients by the tertiles of both the maximal ACh-stimulated forearm blood flow (FBF) and HOMA (**a**), and crude (**b**) incident of events by the tertiles of endothelium-dependent vasodilation and HOMA.

The patients have been followed up for a median period of 117 months (range: 31–211), during which we recorded 223 new cases of HF (3.3 events/100 patient-years, 111 cases had an ischemic etiology, 20 were attributable to diabetic cardiomyopathy, and 92 had a hypertensive etiology). Considering the HF classification based on ejection fraction, we documented 145 cases of HF with preserved ejection fraction (HF-pEF) and 78 cases of HF with reduced EF (HF-rEF); the patients with HF-rEF were older (53.8 ± 9.4 vs. 48.1 ± 8.9 years; *p* < 0.0001) with a lower percentage of females (41.0 vs. 58.9%). We reported event-free survival curves according to the tertiles of the maximal ACh-stimulated FBF mean value, as shown in Figure 1. On the right side of the same figure, we have graphed the crude rate of HF occurrence by both the tertiles of the maximal ACh-stimulated FBF and HOMA. 

**Figure 2:**
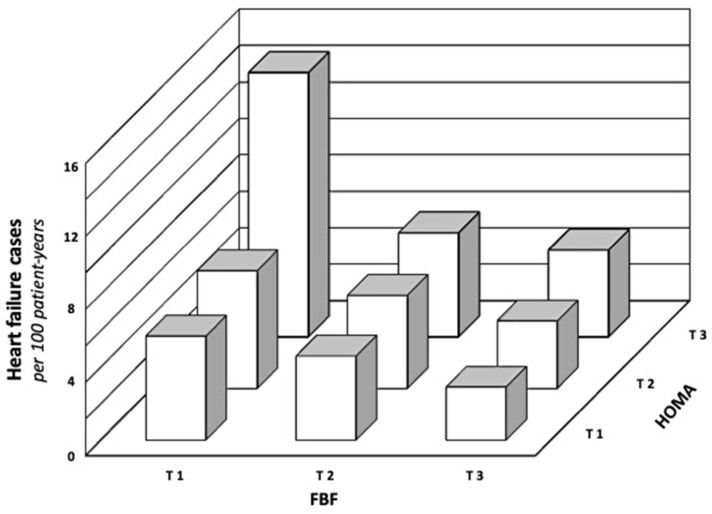
Rate of heart failure events in relation to the tertiles of HOMA and endothelium-dependent vasodilation, demonstrating a clear interaction between them.

Figure 2 shows the incident HF cases by the tertiles of both ACh-stimulated FBF and HOMA; for every tertile of ACh-stimulated FBF, the rate of total events significantly increases from the first to the third tertile of HOMA (log-rank test; all *p* < 0.0001). 

### 3.3. Cox Regression Analyses

In univariate analysis (Table 2), the incident risk of HF was directly related to hs-CRP (HR = 1.451, 95% CI = 1.309–1.608), HOMA (HR = 1.334, 95% CI = 1.213–1.468), and age (10 years increase, HR = 1.193, 95% CI = 1.030–1.381), and was inversely related to the maximal ACh-stimulated FBF [100% increase, HR = 0.660, (95% CI = 0.585–0.744)], e-GFR [(10 mL/min/1.73 m^2^ increase, HR = 0.538, 95% CI 0.484–0.599)]. The female gender increases the risk of incident HF more than doubled (HR = 2.303, 95% CI = 1.661–3.183). No association was found between the occurrence of HF and SBP, BMI, smoking, and total cholesterol.

In the multivariate Cox regression analysis (Table 2), including the variables reaching the statistical significance at univariate analysis, serum hs-CRP (HR = 1.362, (95% CI = 1.208–1.536), HOMA (HR = 1.293, 95% CI = 1.142–1.465), the maximal ACh-stimulated FBF (100% of increase, HR = 0.807, (95% CI = 0.697–0.934), and e-GFR (10 mL/min/1.73 m^2^ increase, HR = 0.552, 95% CI = 0.483–0.603) maintained an independent association with incident HF (Table 2). Interestingly, both age and gender were not retained in the final model. 

### 3.4. Mutual Effect Modification by ACh-Stimulated FBF and HOMA

A possible mutual effect modification between HOMA and the maximal ACh-stimulated FBF in predicting incident HF was investigated in crude and adjusted linear regression models: Model 1 (including FBF, HOMA, and their interaction term) and Model 2 (including the above-mentioned variables in addition to age, gender, smoking, SBP, BMI, cholesterol and anti-hypertensive drugs). The results of both crude and adjusted models demonstrated that HOMA significantly modified the effect of ACh-stimulated FBF on incident HF and, given the mutualistic nature of the effect modification, ACh-stimulated FBF also significantly changed the effect of HOMA on HF development (Table 3, Models 1 and 2).

Successively, we performed another analysis stratifying the study population into four groups based on the median of both the maximal ACh-stimulated FBF and HOMA, which demonstrated a competitive interaction between the two variables in predicting the risk of incident HF (Table 4). In fact, in the absence of competitive interaction between FBF and HOMA, the expected risk in patients with the least favorable median of the two variables would be equal to or greater than the sum of the single risk versus that of the reference group. However, the observed risk of patients in Group 3 (with FBF median < 252% and HOMA median ≥ 3.1) versus the reference group was much lower than expected, confirming a competitive interaction between the two risk factors.

## 4. Discussion

The major finding obtained in the present study, conducted in a very large population of well-characterized never treated hypertensive patients, is the demonstration, for the first time, of a possible competitive interaction between the maximal ACh-stimulated FBF and HOMA in the appearance of incident HF. The biological plausibility of this evidence is based on the fact that our data demonstrate that HOMA significantly modifies the prognostic effect of ACh-stimulated FBF on incident HF and vice versa. On this basis, it is evident that, as reported in Table 3, the less favorable median value of one of the two variables significantly reduces the risk of HF attributable to the other variable. 

Obviously, the presence of the mutual effect modification should not diminish the importance of the other evidence (clinically and prognostically relevant) highlighted by the results of the present study. Firstly, the evidence that endothelial dysfunction can predict HF appearance is another important finding that allows us to expand previously published data that demonstrated that endothelial dysfunction is a consequence of HF [13,14,15]. Thus, our findings reinforce the causative role of endothelial dysfunction in the cardiovascular continuum from hypertension to clinical outcomes, as already demonstrated by our group [12] in the same population. Secondly, for the first time, we demonstrated that insulin resistance (evaluated by HOMA) is an independent and strong predictor of incident HF; this finding, obtained in a very well-characterized population, represents an additional step in the comprehension of the pathophysiological mechanisms shared by both HF and insulin resistance in the continuum of cardio-metabolic diseases. This evidence is particularly relevant because the insulin resistance status is very frequent in hypertensive patients and is very often unrecognized and scarcely investigated. The hyperactivity of the sympathetic nervous system [19] with associated major renal reabsorption of sodium and water may be the main pathogenetic mechanism involved in HF appearance. In addition, the anabolic properties of insulin and its interplay with both growth hormone and IGF-1 may contribute to the increase of cardiac mass, as previously demonstrated by us [20,21]. Furthermore, the close relationship between insulin resistance and endothelial dysfunction is of no less clinical and pathogenetic significance in HF development and progression. 

Of note, it is important to underline that insulin resistance is a metabolic alteration that precedes many years before the appearance of overt diabetes, thus conferring to our evidence of an important clinical and prognostic significance because it allows us to identify subjects at risk in a very early step of the disease natural history. Furthermore, it is mandatory to remark on the strict bidirectional relationship between insulin resistance and endothelial dysfunction, since they mutually amplify their negative effects in a reverberant vicious circle [11,22]. With regards to the higher females’ prevalence in the progressors group, the biological plausibility may be explained by the recent findings that demonstrated that cardiac glucose metabolism of insulin-resistant females is significantly reduced in comparison with men, as a consequence of a specific and early myocardial insulin-resistance [23,24].

Finally, an excess of oxidative stress that reduces NO bioavailability, as well as the following neurohormonal activation and release of inflammatory mediators, may be hypothesized as other possible pathogenetic mechanisms involved in the appearance and progression of HF [14]. Furthermore, it is clearly demonstrated that all these factors increase arterial stiffness, which represents another important pathogenetic mechanism linking endothelial dysfunction to HF appearance [25,26]. In keeping with this, we previously reported that endothelial dysfunction in essential hypertension is inversely related to pulse pressure (i.e., a marker of vascular aging and arterial stiffness) [27]. 

## 5. Conclusions

In conclusion, it is possible to affirm that both endothelial dysfunction (evaluated by strain-gauge plethysmography) and insulin resistance (measured by HOMA) are independent and strong predictors of incident HF in never treated hypertensive patients. In addition, our data demonstrate that these two conditions interact with each other with a competitive mechanism. Moreover, given the bidirectional mechanism between endothelial dysfunction and insulin resistance [2,11] and their interaction in promoting structural and functional cardiac damage, it is reasonable to reinforce the need to optimize the risk stratification strategies of hypertensive patients through careful phenotyping to modify the cardiovascular continuum from essential hypertension to HF and death. 

The strengths of this study are (1) that we directly tested endothelial function by stimulating muscarinic cholinergic receptors by intra-arterial infusion of vasoactive agonist in a very large and well-characterized population, and (2) the follow-up duration. 

On the contrary, the major limitation of the study is the invasiveness (even if minimal) of the strain-gauge plethysmography.

## Figures and Tables

**Table 1:** Baseline characteristics of the study population stratified into progressors and non-progressors to heart failure.

	All (n = 705)	Progressors (n = 223)	Non-Progressors (n = 482)	*p*
Gender, female (%)	347 (49.2)	141 (63.2)	206 (42.7)	0.0001
Age, years	48.4 ± 10.6	49.8 ± 11.0	47.8 ± 10.3	0.019
BMI, Kg/m^2^	27.5 ± 3.6	27.8 ± 4.1	27.3 ± 3.4	0.089
Current smokers, n (%)	110 (15.6)	38 (17.0)	72 (14.9)	0.237
Systolic BP, mmHg	149.2 ± 16.9	150.3 ± 16.1	148.7 ± 17.3	0.243
Diastolic BP, mmHg	91.0 ± 11.6	90.9 ± 11.0	91.0 ± 11.8	0.914
Heart rate, bpm	72.6 ± 9.6	70.8 ± 9.2	73.4 ± 9.7	0.437
Fasting glucose, mg/dL	95.2 ± 10.6	96.8 ± 11.1	94.4 ± 10.2	0.004
Fasting insulin, U/L	14.9 ± 6.9	16.6 ± 7.1	13.4 ± 6.1	0.0001
HOMA	3.4 ± 1.7	4.0 ± 1.9	3.1 ± 1.5	0.0001
Total cholesterol, mg/dL	204.7 ± 31.6	203.7 ± 32.3	205.2 ± 31.5	0.559
LDL cholesterol	129.3 ± 31.5	128.9 ± 32.4	129.4 ± 31.1	0.844
HDL cholesterol	51.8 ± 12.4	50.8 ± 12.8	52.2 ± 12.2	0.163
Triglyceride, mg/dL	115.9 ± 39.2	117.1 ± 40.4	115.5 ± 38.7	0.614
Creatinine, mg/dL	0.96 ± 0.19	1.08 ± 0.19	0.90 ± 0.16	0.0001
e-GFR, mL/min/1.7 m^2^	84.6 ± 20.2	70.1 ± 17.6	91.2 ± 17.7	0.0001
hs-CRP, mg/dL	3.75 ± 1.68	4.45 ± 1.39	3.43 ± 1.71	0.0001
***Forearm blood flow***				
Basal, mL × 100 mL tissue^−1^ × min^−1^	3.36 ± 0.66	3.29 ± 0.61	3.37 ± 0.67	0.130
Acetylcholine, % increase	294 ± 179	221 ± 128	328 ± 189	0.0001
Sodium nitroprusside, % increase	316 ± 112	312 ± 114	318 ± 111	0.584
***Antihypertensive drugs***				
ACE-i/ARBs, n (%)	558 (79.1)	175 (78.5)	383 (79.5)	0.382
Calcium antagonists, n (%)	248 (35.2)	78 (34.9)	170 (35.3)	0.469
β-Blockers, n (%)	57 (8.1)	19 (8.5)	38 (7.9)	0.386
α-Blockers, n (%)	15 (2.1)	5 (2.2)	10 (2.1)	0.443
Diuretics, n (%)	126 (17.9)	40 (17.9)	86 (17.8)	0.487
Associations, n (%)	408 (57.8)	128 (57.4)	280 (58.1)	0.431

ACE-I = angiotensin converting enzyme inhibitors; ARBs = angiotensin receptor blockers; BMI = body mass index; BP = blood pressure; e-GFR = estimated glomerular filtration rate; HDL = high density lipoprotein; HOMA = Homeostasis model assessment; hs-CRP = high-sensitivity C-reactive protein; and LDL = low density lipoprotein.

**Table 2:** Univariate and multivariate Cox regression analyses for incident heart failure.

*Univariate Analysis*	Hazard Ratio	95% CI	*p*
Gender, female	2.303	1.661–3.193	0.0000
hs-CRP, mg/dL	1.451	1.309–1.608	0.0000
HOMA	1.334	1.213–1.468	0.0000
Age, 10 years	1.193	1.030–1.381	0.017
Forearm blood flow, 100% increase	0.660	0.585–0.744	0.0000
e-GFR, 10 mL/min/1.7 m^2^	0.538	0.484–0.599	0.0000
Systolic BP, 10 mmHg	1.054	0.961–1.156	0.260
BMI, Kg/m^2^	1.037	0.993–1.083	0.099
Smoking	1.169	0.761–1.797	0.474
Total cholesterol, 10 mg/dL	0.977	0.929–1.027	0.368
***Multivariate analysis***			
hs-CRP, 1 mg/dL	1.362	1.208–1.536	0.00001
HOMA	1.293	1.142–1.465	0.0001
Forearm blood flow, 100% increase	0.807	0.697–0.934	0.004
e-GFR, 10 mL/min/1.7 m^2^	0.552	0.483–0.630	0.00001

BMI = body mass index; BP = blood pressure; e-GFR = estimated glomerular filtration rate; HOMA = Homeostasis model assessment; and hs-CRP = high-sensitivity C-reactive protein.

**Table 3:** Cox regression analysis of the mutual effect modification by FBF and HOMA on the incidence of heart failure. Dependent variable: heart failure.

	Model 1	Model 2 *
**HOMA < 3.11**	HR = 3.52, 95% CI: 2.20–5.63; *p* < 0.001 (*FBF ≤ 252% versus FBF > 252%*)	HR = 3.95, 95% CI: 2.46–6.34; *p* < 0.001 (*FBF ≤ 252% versus FBF > 252%*)
**HOMA ≥ 3.1**	HR 1.60, 95% CI: 1.11–2.33; *p* = 0.012 (*FBF ≤ 252% versus FBF > 252%*)	HR = 1.72, 95% CI: 1.18–2.50; *p* = 0.004 (*FBF ≤ 252% versus FBF > 252%*)
	**Effect modification by HOMA** ***p* = 0.01**	**Effect modification by HOMA** ***p* = 0.007**
**FBF ≥ 252%**	HR = 2.57, 95% CI: 1.56–4.22; *p* < 0.001 (*HOMA > 3.1 versus HOMA ≤ 3.1*)	HR = 2.68, 95% CI: 1.61–4.45; *p* < 0.001 (*HOMA > 3.1 versus HOMA ≤ 3.1*)
**FBF < 252%**	HR = 1.17, 95% CI: 0.84–1.64; *p* = 0.35 (*HOMA > 3.1 versus HOMA ≤ 3.1*)	HR = 1.17, 95% CI: 0.83–1.65; *p* = 0.38 (*HOMA > 3.1 versus HOMA ≤ 3.1*):
	**Effect modification by FBF, *p* = 0.007**	**Effect modification by FBF, *p* = 0.007**

CI = confidence interval; FBF = forearm blood flow; HOMA = homeostasis model assessment; and HR = hazard ratio. * Adjusted for age, gender, smoking, systolic BP, body mass index, cholesterol, and antihypertensive drugs. HOMA median = 3.1, ACh-stimulated FBF median = 252%.

**Table 4:** Interaction analysis between the maximal ACh-stimulated FBF and HOMA in predicting incident heart failure.

	HR	CI 95%	*p*
0	1		
1	3.625	2.076–6.331	0.0001
2	5.938	3.459–10.193	0.0001
3	6.548	4.034–10.629	0.0001

CI = confidence interval; HR = hazard ratio. 0: HOMA under the median; and ACh-stimulated FBF above the median (reference group). 1: HOMA above the median; ACh-stimulated FBF above the median. 2: HOMA under the median; ACh-stimulated FBF under the median. 3: HOMA above the median; and ACh-stimulated FBF under the median.

## Data Availability

The data that support the findings of this study are available on request from the corresponding author.
